# The Emerging Role and Clinical Significance of PI3K-Akt-mTOR in Rhabdomyosarcoma

**DOI:** 10.3390/biom15030334

**Published:** 2025-02-25

**Authors:** Ilaria Versari, Sara Salucci, Alberto Bavelloni, Michela Battistelli, Mirko Traversari, Ashley Wang, Maurilio Sampaolesi, Irene Faenza

**Affiliations:** 1 Department of Biomedical and NeuroMotor Sciences (DIBINEM), University of Bologna, 40126 Bologna, Italy; ilaria.versari4@unibo.it (I.V.); sara.salucci@unibo.it (S.S.); 2 Laboratory of Experimental Oncology, IRCCS, Istituto Ortopedico Rizzoli, 40136 Bologna, Italy; alberto.bavelloni@ior.it; 3 Department of Biomolecular Sciences, University of Urbino Carlo Bo, 61029 Urbino, Italy; michela.battistelli@uniurb.it; 4 Department of Medical and Surgical Sciences (DIMEC), University of Bologna, 40126 Bologna, Italy; mirko.traversari2@unibo.it; 5 Translational Cardiomyology Laboratory, Stem Cell Biology and Embryology, Department of Development and Regeneration, KU Leuven, 3000 Leuven, Belgium; ashley.wang@kuleuven.be (A.W.); maurilio.sampaolesi@kuleuven.be (M.S.)

**Keywords:** rhabdomyosarcoma, PI3K, Akt, mTOR

## Abstract

Rhabdomyosarcoma (RMS) is a common soft tissue sarcoma primarily affecting children and young adults. This disease is more prevalent in children under 15, with two main types: embryonal Rhabdomyosarcoma (eRMS), which has a better prognosis, and alveolar Rhabdomyosarcoma (aRMS), which is more aggressive and associated with specific genetic alterations. The PI3K-Akt-mTOR pathway is often hyperactivated in RMS, contributing to cell proliferation, survival, and resistance to therapies. The presence of phosphorylated components of this pathway correlates with poor survival outcomes. Here, we discuss various therapeutic approaches targeting the PI3K-Akt-mTOR pathway. These include the use of specific inhibitors (e.g., PI3K inhibitors, Akt inhibitors) and combination therapies that may enhance treatment efficacy. Dietary supplements like curcumin and repurposed drugs such as chloroquine are also mentioned for their potential to induce apoptosis in RMS cells. We also emphasize the need for innovative strategies to improve survival rates, which have remained stagnant over the years. Targeting super-enhancers and transcription factors associated with RMS may provide new therapeutic avenues. Overall, this review underscores the critical role of the PI3K-Akt-mTOR pathway in RMS and the potential for targeted therapies to improve patient outcomes.

## 1. Introduction: Rhabdomyosarcoma Features

**Figure 1:**
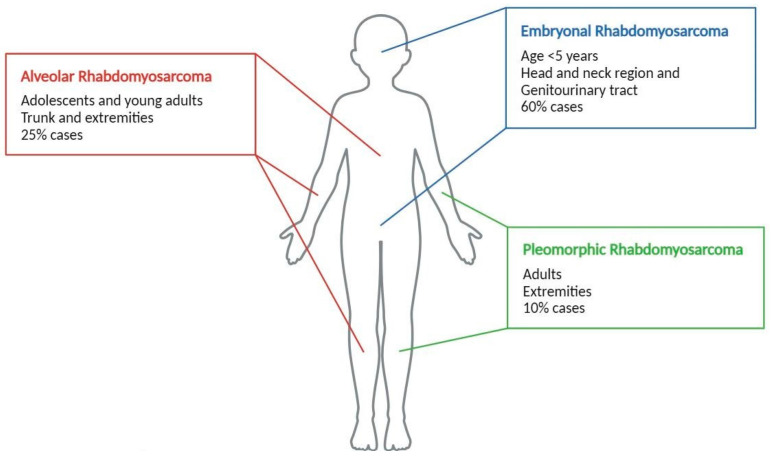
A schematic representation of the main rhabdomyosarcoma histotypes and their clinical features.

Rhabdomyosarcoma (RMS) is a common form of soft tissue sarcoma, mainly diagnosed in children and young adults. It represents approximately 50% of pediatric soft tissue sarcomas, and the majority of RMS patients are younger than 15 years old [1]. The overall 5-year survival rate for RMS is about 25%, with adults showing a poorer prognosis [2], whereas patients who are 1-to-9 years old are more likely to survive [3]. Additional risk is posed by a tumor size exceeding 5 cm in diameter, tumor invasiveness, and metastasis [4]. RMS shows features of the skeletal muscle lineage (i.e., myogenic markers, such as desmin, myosin, myoglobin, actin) [5], and indeed, it originates from mesenchymal cell precursors with impaired differentiation ability [5]. In a murine model, the combination of muscular dystrophy and aging further increases the risk of RMS. An aged microenvironment is less effective at maintaining the myogenic fate of muscle stem cells, leading to a shift towards fibrogenic and adipogenic differentiation, which may promote tumor development [6]. However, in humans, this correlation has not been proven yet. The latest classification of RMS is primarily based on histological features and genetic markers and describes two predominant histotypes (Figure 1): embryonal rhabdomyosarcoma (eRMS) and alveolar rhabdomyosarcoma (aRMS), both associated with a round-cell phenotype. Other less common subtypes show spindle cell, epithelioid, or pleomorphic phenotypes and may be further defined by genetic alterations [7]. eRMS is the most common variant, accounting for 60% of the cases, whereas aRMS represents 25% of diagnoses [8,9]. Embryonal RMS typically arises in the head and neck region or in the genitourinary tract and is frequently diagnosed in children younger than five [7]. Single driver mutations are not known for this histotype, but the merging of many copy number variations and alterations of the RAS pathway originates eRMS [7]. Gains of chromosomes 2, 8, and 12 have also been described in eRMS [10]. Alveolar RMS usually involves the extremities and the trunk and is common in adolescents and young adults [7]. In most cases, the alveolar subtype is related to chromosomal translocations resulting in FOXO1 gene fusion to PAX3 or PAX7, an indicator of a poorer prognosis. From a molecular point of view, RMS is classified primarily based on the presence of specific genetic alterations, particularly chromosomal translocations that lead to the formation of fusion genes. In particular, RMS tumors can be distinguished into fusion-positive and fusion-negative depending on the presence or absence of the PAX3-FOXO1 or the PAX7-FOXO1 fusion products, which result in increased transcriptional activity of genes inducing oncogenic transformation, such as MET, ALK1, MYCN, IGFR1, IGF-2, and FGFR4 [11,12]. Approximately 80% of aRMS cases are fusion-positive [1,13]. Fusion-negative aRMS forms display various alterations of genes that are involved in a restricted number of signaling pathways and the same pathways, such as the PI3K-Akt-mTOR and the RAS-RAF-MAPK axes, are frequently implicated in fusion-positive RMS [1,9]. Moreover, fusion-negative aRMS cases show mutations typical of eRMS tumors [14]. The molecular classification of RMS is important for understanding the underlying mechanisms of tumorigenesis and for developing targeted therapeutic strategies. The PI3K-Akt signaling plays a significant role in the biology of RMS cells, influencing various cellular processes that are critical for tumor growth and progression. In this review, we delve into some key aspects of the PI3K-Akt pathway in the context of rhabdomyosarcoma.

## 2. The PI3K-Akt-mTOR Pathway: Structure, Components, and Activity

PI3K-Akt-mTOR is a signal transduction pathway that has an important role in regulating many cellular processes, including survival, proliferation, and differentiation [15].

Cell surface receptors, triggered by growth factors, cytokines, and other extracellular stimuli, activate the Phosphatidylinositol 3-kinases (PI3Ks), initiators of the signaling cascade. The PI3K family is divided into three classes of kinases, each characterized by substrate specificity, distinct structure and products. Class I is further divided into subclasses A and B, whereas knowledge is limited for classes II and III [16,17]. Class IA PI3Ks are formed by a catalytic subunit (p110) and a regulatory subunit (p85) and are activated by receptor tyrosine kinases (RTKs) or G protein-coupled receptors (GPCRs) [16]. When the system is inactive, p85 interacts with p110. However, when receptors are turned on, PI3K is recruited to the plasma membrane through p85 binding, relieving the catalytic subunit. Hence, p110 is free to phosphorylate Phosphatidylinositol 4,5-bisphosphate (PtdIns(4,5)P2) to Phosphatidylinositol 3,4,5-trisphosphate (PtdIns(3,4,5)P3). Class IB PI3Ks consist of a p110γ catalytic subunit and a p101 regulatory subunit [16]. They are activated by GPCRs, and the generation of PtdIns(3,4,5)P3 is similar to class IA. The lipidic product PtdIns(3,4,5)P3 acts as a docking site for signaling proteins and activates signal transduction pathways governing many cellular processes [16,18], thus its presence is controlled by PTEN phosphatase, which dephosphorylates PtdIns(3,4,5)P3 back to PtdIns(4,5)P2, therefore antagonizing PI3K activity. PtdIns(3,4,5)P3 recruits Akt to the plasma membrane, where it is first phosphorylated by phosphoinositide-dependent protein kinase 1 (PDK1) at T308 site and subsequently by various PDK2 at S473 [19]. Akt, also known as protein kinase B (PKB), is a serine–threonine kinase that mediates mTORC1 phosphorylation and the activation of many other downstream substrates, regulating cell growth and proliferation, survival, motility, angiogenesis, and apoptosis [20,21]. Akt exists in three different isoforms, namely Akt1, Akt2, and Akt3. mTOR is a serine–threonine kinase found in two complexes: mTORC1 and mTORC2. mTORC1 substrates include the translation initiation factor 4EBP1 and the ribosomal protein S6 kinase (S6K1), related to protein synthesis and cellular proliferation. The mTORC2 complex, instead, acts as a PDK2 for the phosphorylation of Akt, resulting in its full activation and leading to phosphorylating events both in the cytoplasm and in the nucleus [16]. The PI3K pathway is frequently upregulated in tumors because of genetic or epigenetic alterations of PI3K isoforms and RTKs or due to the loss of PTEN [17,19,22]. In addition, other components, such as Akt, are sometimes altered in cancer [22].

## 3. The PI3K-Akt-mTOR Pathway in Rhabdomyosarcoma

**Figure 2:**
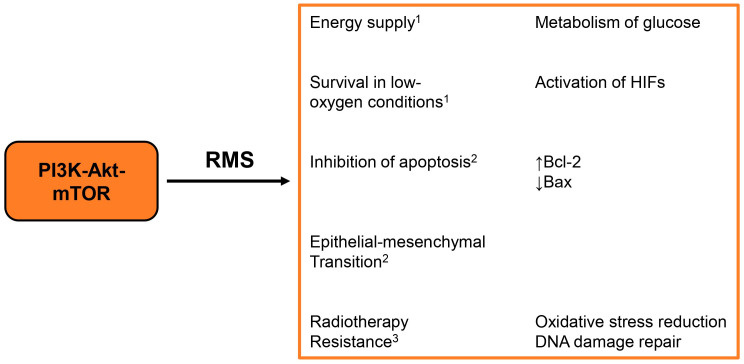
Results of the PI3K-Akt-mTOR activation in rhabdomyosarcoma. The left column describes the main events occurring after the pathway activation; the right column details the mechanisms involved; superscript numbers indicate the references of the relevant studies: 1. E. Monti et al., 2016 [8]; 2. Zhang, Q. et al., 2019 [23]; 3. S. Codenotti et al. [34].

It is known that the PI3K-Akt axis promotes cell proliferation by regulating the cell cycle [15,22]. In rhabdomyosarcoma cells, the activation of this cascade leads to the increased expression of cell cycle regulators, such as cyclin E2 and CDK2, facilitating the progression through the cell cycle and enhancing tumor growth. In RMS, the activation of this pathway can inhibit apoptotic processes, allowing cancer cells to evade programmed cell death (Figure 2). This mainly occurs through the upregulation of anti-apoptotic proteins like Bcl-2 and the downregulation of pro-apoptotic proteins like Bax [23]. PI3K-Akt signaling is also involved in promoting migration and invasiveness in cancer cells (Figure 2). As a matter of fact, in RMS, it can facilitate epithelial–mesenchymal transition (EMT), which is associated with increased motility and the ability to invade surrounding tissues [23].

RMS genetic alterations have been investigated by genome sequencing, which revealed a dysregulation of many signaling pathways, including the PI3K-Akt-mTOR. In 2012, Shukla and colleagues identified mutations in 21% of RMS samples out of 89 specimens. Genetic alterations were particularly common in eRMS cases (28%) and, to a lesser extent, in aRMS (3.5%). Modifications of PIK3CA in genomic DNA were identified in 5% of eRMS cases out of 60 eRMS samples, and the authors suggested a role of these activating mutations in triggering Akt [24]. In addition, FGFR4 mutations were identified in 9% of eRMS, but no aRMS, specimens. A genome-wide investigation, conducted in 2014 on 147 RMS samples, found alterations of the RTK-RAS-PIK3CA axis in 93% of rhabdomyosarcoma cases, and in particular, PIK3CA was mutated in 7.4% of fusion-negative samples included in the study [25]. Additional research was conducted by Seki and colleagues, who highlighted the importance of the FGFR4-RAS-Akt pathway, whose genes were mutated in 40% of 60 RMS cases. These alterations were predominantly observed in eRMS [26]. Epigenetic changes of the axis were also investigated in a methylome analysis, which revealed PTEN promoter hypermethylation in 20 out of 22 eRMS cases (90.9%), suggesting a role of the epigenetic silencing of PTEN in embryonal RMS [26]. Modifications of the PI3K-Akt-mTOR status may derive, in fusion-positive cases, from the PAX3-FOXO1 product, which is able to influence its transcriptional targets. PAX3-FOXO1 can also affect the expression of specific miRNAs that regulate the components of the PI3K signaling pathway, further promoting its activation [9]. For instance, the PTEN gene can be downregulated [27], resulting in the increased activity of Akt and downstream proteins. On the contrary, IGF-1R can be upregulated by PAX3-FOXO1 in aRMS, leading to the proliferation of tumor cells through Akt-mTOR [28]. Codenotti et al. showed that the expression of a constitutively active form of Akt (myrAkt1) promotes tumor growth, cell spread, and DNA repair in the human eRMS RD cell line. They also highlighted that oncogenic Akt1 signaling originates conspicuous cellular susceptibility to 2-Deoxy-D-Glucose (2-DG) and lovastatin, indicating that hyperactivated Akt1 induces RMS aggressiveness in the presence of glucose pathway metabolites and mevalonate [29]. Moreover, FGFR4 is also commonly altered both in aRMS, being a transcriptional target of PAX3-FOXO1, and in eRMS, due to activating mutations [30]. FGFR4 is the most mutated RTK in fusion-negative RMS [31], and when stimulated, it activates the PI3K-Akt-mTOR cascade [32]. As reviewed by Monti and Fanzani, the PI3K-Akt-mTOR axis influences the metabolism of glucose and other nutrients (Figure 2), providing the energy supply for migration and metastasis in RMS [8]. The pathway is also involved in tumor survival in low oxygen conditions (Figure 2) due to the activation of Hypoxia Inducible Factors (HIFs) [8]. In addition, the presence of phosphorylated components of the pathway has been associated with poor disease-free and overall survival of RMS patients [33], and the hyperactivation of downstream components of Akt signaling is related to radiotherapy resistance through oxidative stress reduction and cell survival, as it was observed in RD cell line [34]. In fact, the Akt1/Src/Caveolin-1 (Cav1) signaling pathway is activated in response to high oxidative stress and enhances the ability of RMS cells to detoxify reactive oxygen species (ROS) and repair DNA damage (Figure 2). Increased resistance to oxidative stress contributes to radioresistance, making it more challenging to treat RMS effectively with standard therapies. Codenotti et al. claimed that statins induce oxidative stress in RMS cells and disrupt the protective mechanisms that cancer cells employ against oxidative damage, therefore improving the success of radiotherapy [34]. In another study, the PI3K-Akt signaling pathway was demonstrated to play a crucial role in mediating the effects of perfluorooctanoic acid (PFOA) on RD cells [23]. PFOA is considered a persistent organic pollutant, meaning that it does not break down easily and can accumulate in the human body and in the environment. Overall, the study suggests that the activation of the PI3K-Akt signaling pathway by PFOA is central to its effects on cell proliferation, migration, invasion, and apoptosis in RD cells, highlighting its potential role in cancer development [23].

## 4. Treatments Targeting the Pathway in RMS

**Figure 3:**
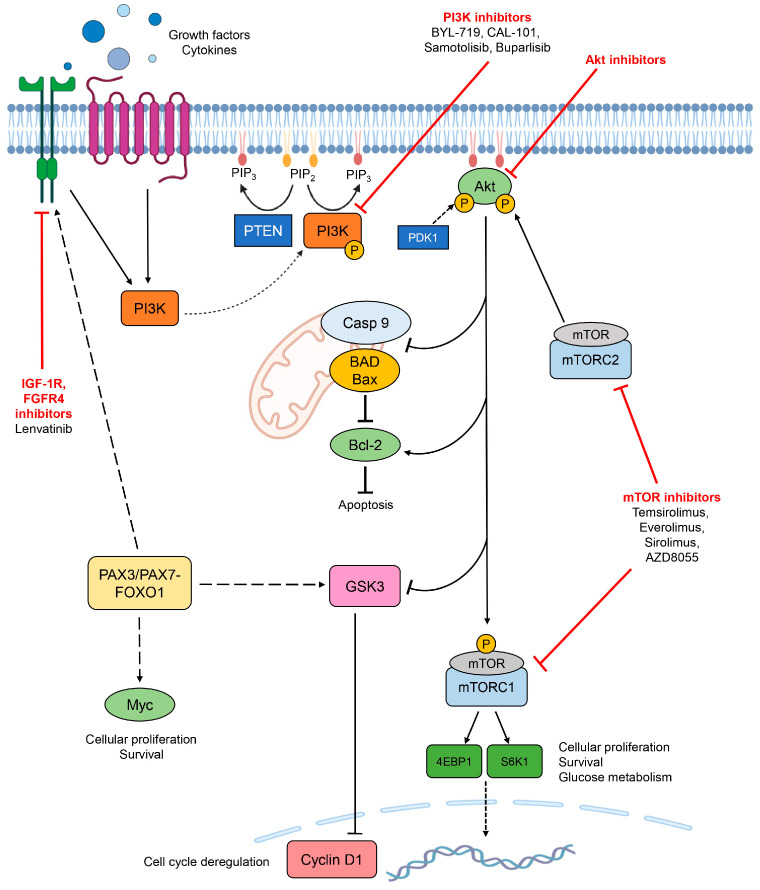
A schematic representation of the PI3K-Akt-mTOR pathway with its fundamental components and the activation of the main cellular responses in rhabdomyosarcoma. Red arrows show the action of direct inhibitors of the PI3K-Akt-mTOR pathway, and the names of some drugs are reported as an example. In the context of rhabdomyosarcoma, BYL-719, CAL-101, Buparlisib, and AZD8055 are in preclinical trials; Everolimus, Lenvatinib, Samotolisib, Sirolimus, and Temsirolimus are in clinical trials. Abbreviations: PIP_2_, Phosphatidylinositol 4,5-bisphosphate; PIP_3_, Phosphatidylinositol 3,4,5-trisphosphate.

Current treatments for RMS patients are based on surgery, radiotherapy, and a three-drug chemotherapy with vincristine, actinomycin D, and cyclophosphamide [11]. However, in some cases, the prognosis is still poor, and chemotherapy shows little activity in metastatic cases [11]. For these reasons, innovative approaches are needed. Targeted therapies have been demonstrated to be successful in the treatment of many types of cancer due to the identification of specific signature and signaling networks of a certain tumor. The deregulation of cell growth and survival, caused by the activation of PI3K signaling, contributes to competitive growth advantage and often to therapeutic resistance [19]. Therefore, this pathway is an interesting target for the development of new anticancer agents. As a matter of fact, many inhibitors targeting key components of the pathway (Figure 3) are being developed by pharmaceutical companies and are currently under preclinical and clinical trials [16]. These targeted therapies can be divided into four main groups, based on the selected component of the cascade: PI3K inhibitors, dual PI3K-mTOR inhibitors, mTOR inhibitors, and Akt inhibitors. In addition, other molecules may be used for interfering with the pathway, for instance, PDK1 or PtdIns analogues [35].

Inhibitors of PI3K can be divided into isoform-specific inhibitors, which target catalytic subunits of a single isoform, and pan-PI3K inhibitors, which are directed towards all class IA PI3Ks [22]. These compounds may block the catalytic activity of PI3K by different mechanisms: irreversible inhibition or ATP-competitive modulation [35]. Considering that distinct PI3K isoforms have different roles, isoform-specific inhibitors may be more effective. In addition, the complete inhibition of all isoforms, deriving from the usage of non-selective inhibitors, may cause toxicity [22,23]. However, isoform-specific PI3K inhibitors alone exhibit limited efficacy and side effects. Therefore, the combination of different PI3K inhibitors may improve their activity and restrict toxicity. Preclinical studies showed that the combination of BYL-719 and CAL-101 (both class I PI3K inhibitors) leads to a greater reduction in cell viability and to increased apoptosis in RMS cell lines compared to either agent alone, thus suggesting a potential synergistic effect that could improve treatment outcomes [36]. Some PI3K inhibitors are also able to block mTOR since these components share similar structures. The concurrent inhibition of multiple elements of the pathway may result in higher efficacy, especially when PI3K is not the only regulator of the mTOR complex [22]. Furthermore, the simultaneous targeting of two kinases decreases the likelihood of therapeutic resistance [16]. Rapamycin and its analogues are first-generation allosteric agents that can effectively inhibit mTORC1, but not mTORC2, by direct binding. However, it is established that the suppression of complex 1 by rapamycin and its derivatives induces a feedback activation of PI3K and Akt [37] that may be reduced by using dual PI3K-mTOR inhibitors [22]. On the other hand, second-generation mTOR catalytic site inhibitors, which are mainly ATP-competitive, directly target mTOR and, in turn, block both complexes 1 and 2 [22,38] and suppress downstream effectors [21]. Akt inhibitors can be ATP mimetics, which block the catalytic site, or allosteric inhibitors, which interfere with the binding to the PtdIns, and therefore stop Akt recruitment to the membrane [22]. Most ATP-competitive inhibitors are non-selective, and they target all three Akt isoforms. Akt1/2 dual inhibitors or pan-Akt inhibitors, targeting isoforms 1, 2, and 3, have been demonstrated to be more effective [39] because of the distinct roles of the isoforms.

Some agents targeting the PI3K-Akt-mTOR pathway have already been approved by the FDA for the treatment of other conditions, but their usage and limitations are ambiguous. For instance, idelalisib, copanlisib, duvelisib, and umbralisib (all PI3K inhibitors) received an accelerated approval for hematological malignancies, but subsequent post-approval trials led to safety alerts and limitations of use due to potential toxicity and severe side effects [40,41]. Other inhibitors are currently under clinical trials, where their safety and efficacy are being tested on RMS (Table 1). For example, a recent study discussed a randomized, open-label, phase III clinical trial (ARST1431) from the Children’s Oncology Group (COG) regarding the treatment of intermediate-risk rhabdomyosarcoma, focusing on combining standard chemotherapy with temsirolimus, an mTOR inhibitor [42]. This treatment did not improve event-free survival or overall survival despite the promising preclinical and clinical data obtained previously [43]. One possible explanation could be the lower number of aRMS patients enrolled in the phase III trial compared to the phase II [44]. Notably, not any of the recent clinical trials (Table 1) proved the effectiveness of the tested drugs on RMS patients, therefore suggesting a more personalized treatment approach based on genetic testing [42,45].

**Figure 4:**
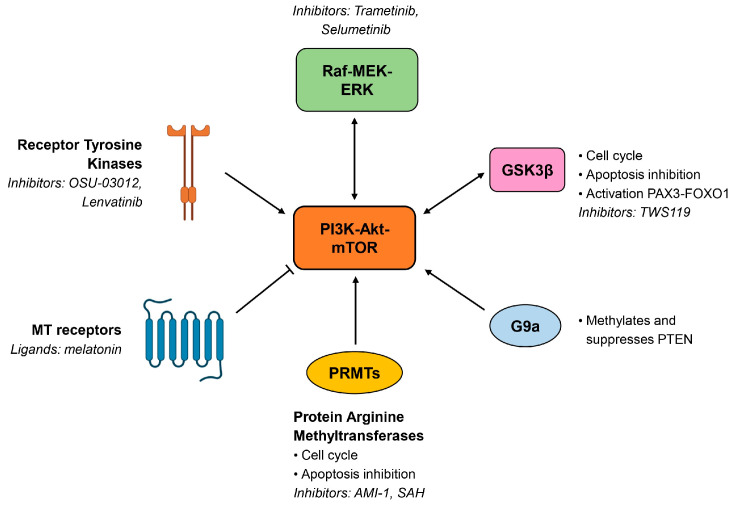
Interactions of the PI3K-Akt-mTOR pathway with other signaling pathways and components. The Raf-MEK-ERK pathway and GSK3β are connected with the PI3K-Akt-mTOR pathway in a reciprocal activation; G9a, protein arginine methyltransferases, and receptor tyrosine kinases unilaterally activate the PI3K-Akt-mTOR pathway; MT receptors inhibit the PI3K-Akt-mTOR pathway. Some inhibitors are reported as an example. In the context of RMS, combination therapies of these drugs with PI3K-Akt-mTOR inhibitors are in preclinical trials.

Besides inhibitors that directly target key components of the PI3K-Akt-mTOR pathway (Figure 3), other compounds have been demonstrated to act on this cascade in an indirect manner (Figure 4). Recent research in the cellular biology of rhabdomyosarcoma indicates that abnormal levels of protein arginine methyltransferases (PRMTs) can alter the activity of oncogenes and tumor suppressor genes, leading to neoplastic transformation. Specifically, increased PRMT levels can disturb the cell cycle and inhibit programmed cell death, contributing to cancer progression [46]. Janisiak and colleagues demonstrated that the use of two broad-spectrum PRMT inhibitors, i.e., AMI-1 and SAH effectively reduced the viability, proliferation, and clonogenicity of RMS cells. The inhibitors were shown to decrease the invasive phenotype of RMS cells by reducing their proliferation rate and inducing apoptosis. The inhibitors also attenuated the activity of the PI3K-Akt signaling pathway, suggesting a potential role in treating RMS. By targeting this pathway, the inhibitors may help to control the growth and spread of RMS, making them promising candidates for further research and development in cancer therapy [46].

Considering the role of RTKs in RMS, targeting these receptors and their downstream signaling pathways represents a potential therapeutic approach. Inhibitors of RTKs, e.g., those targeting IGF-1R or FGFR4, have been explored in preclinical models and may enhance the efficacy of existing treatments [28]. Additionally, the identification of RTK expression patterns in RMS could help in stratifying patients for targeted therapies. Studies have indicated that RMS cells, particularly those expressing PAX3-FOXO1, exhibit specific sensitivities to inhibitors of RTKs and their downstream effectors. This suggests that targeting RTK signaling could be an effective approach in treating RMS, especially in combination with other therapies that consider different aspects of tumor biology [28]. OSU-03012 is a small-molecule compound that has been investigated for its potential therapeutic effects in various cancers, including RMS. It is known to act as an inhibitor of the insulin-like growth factor-1 receptor (IGF-1R) and was shown to have anti-tumor activity through multiple mechanisms [47]. In the context of RMS, OSU-03012 has been studied for its ability to inhibit cell proliferation and to induce apoptosis in RMS cell lines. Moreover, OSU-03012 was demonstrated to have effects on the PDK1-Akt axis, further contributing to its anticancer properties. By targeting this pathway, OSU-03012 can potentially enhance the sensitivity of RMS cells to other treatments and overcome resistance mechanisms [47]. In contrast to RTKs, which mediate PI3K-Akt-mTOR activation, MT receptors are able to attenuate the axis. In fact, melatonin was demonstrated to inhibit the pathway by binding to MT2 receptors in several cellular models [48,49], suggesting a rationale for the anticancer effects of melatonin in rhabdomyosarcoma. Indeed, apoptosis and decreased cell proliferation could be observed in RMS cells, which have been shown to express high levels of MT receptors, after the administration of melatonin [50,51].

G9a, also known as EHMT2 (Euchromatic Histone Methyltransferase 2), is a histone methyltransferase that has been found to be overexpressed in several types of tumors, including aRMS. G9a is significant in aRMS since it was identified as an overexpressed and druggable target that plays a critical role in tumorigenesis [52]. Bhat and colleagues claimed that G9a regulates key signaling pathways sustaining tumor growth and proliferation. In particular, G9a represses the expression of the tumor suppressor PTEN in a methylation-dependent manner, which leads to the increased activity of Akt and RAC1, both of which are important for oncogenic signaling in aRMS. Therefore, targeting G9a presents a promising therapeutic strategy for treating aRMS by disrupting its oncogenic functions and promoting the differentiation of tumor cells [52].

Tumor cell adaptation and the acquisition of resistance represent significant limitations of targeted therapies. For this reason, identifying associations with other signaling pathways could bring an opportunity for successful treatment. The inhibition of PI3K may lead to the activation of other pro-survival and growth pathways due to feedback mechanisms [53]. The PI3K–Akt and the Raf–MEK–ERK pathways both promote cell growth and survival and they converge on critical downstream targets such as mTORC1 and the BH3 family of proteins that regulate apoptosis. When one pathway is inhibited, the other may compensate, leading to continuous cell survival and growth. Therefore, combining inhibitors of both the PI3K and MEK axes may be necessary to effectively shut down mTORC1 signaling and promote apoptosis [53]. Indeed, it has been demonstrated that therapies are more effective when drugs targeting the PI3K-Akt-mTOR pathway are combined with inhibitors of other pathways, such as RAS-MEK-ERK [22,53,54]. Combination treatments involving the PI3K inhibitor buparlisib and the MEK inhibitor trametinib showed synergistic effects in preclinical studies [18]. Additionally, the mTOR inhibitor AZD8055 exhibited enhanced efficacy when combined with the MEK inhibitor selumetinib, leading to reduced downstream protein activity in vivo [53]. Dual inhibition represents a strategic therapeutic approach in RMS, potentially leading to improved outcomes by effectively targeting the complex signaling networks that sustain tumor growth and survival. However, there are concerns regarding increased toxicity when using dual-targeting strategies against both PI3K and MEK pathways in patients with advanced cancer. Hence, combining mTOR inhibitors with traditional chemotherapy is considered a more feasible approach, with a better tolerance in pediatric and young adult patients [55].

Akt and GSK3β (Glycogen Synthase Kinase 3 beta) inhibitors are being explored as potential therapeutic options for children and adolescents with various types of cancer, including RMS. Pearson et al. discussed and emphasized the need for rational combination therapies based on the molecular landscape of the tumors. In rhabdomyosarcoma, understanding the specific mutations and alterations present could guide the use of PI3K, mTOR, and Akt inhibitors in combination with other treatments, such as chemotherapy or immunotherapy. Akt and GSK3β inhibitors represent promising avenues for targeted therapy in children and adolescents with cancer. In fact, GSK3β is involved in various cellular processes, including cell cycle regulation, apoptosis, and differentiation [56]. Moreover, GSK3 was found to activate PAX3-FOXO1 fusion protein by phosphorylation [57]. Research on GSK3β inhibitors in pediatric cancers is still emerging but there is growing interest in their potential to improve treatment outcomes, particularly in cancers where GSK3β signaling is disrupted. GSK3β inhibitors such as TWS119 hold promise as a potential therapeutic option for RMS, particularly in the context of combination therapies and targeted approaches [57].

## 5. Future Perspectives

Despite multifactorial approaches, survival rate for RMS has remained quite unwavering over the past decades [58]. Thus, innovative strategies are being evaluated. Dietary supplements, such as curcumin, have been demonstrated to suppress cell migration and to induce cell cycle arrest and apoptosis in RMS cells through the inhibition of mTORC1 at low concentrations and mTORC2 at high concentrations [59,60,61]. Curcumin has the potential to inhibit the Akt signaling pathway in RMS, leading to reduced cell proliferation and increased apoptosis. In addition, changes in the phosphorylation of Akt were observed in some cell lines after curcumin treatment [61]. Another approach consists of repurposing drugs that are currently used as treatments for other pathologies. For example, it has been demonstrated that chloroquine, widely used as anti-malaria drug, synergizes with dual PI3K-mTOR inhibitor NVP-BEZ235 and induces apoptosis in eRMS [62]. Also dihydroartemisinin (DHA), originally used to treat malaria, showed inhibitory activity towards mTORC1 through indirect mechanisms and could be repurposed for RMS [12]. In addition to these approaches, microRNAs (miRNAs) have emerged as key players in RMS biology, providing another avenue for innovative therapeutic strategies [63]. miRNAs are small, non-coding RNAs that regulate gene expression post-transcriptionally, playing crucial roles in cell differentiation, metabolism, and disease progression [64]. They have been demonstrated to regulate fusion-negative RMS, positioning them as novel therapeutic targets [65,66]. Moreover, miR-378 family members are implicated in the Akt-mTOR axis [67], and miR-183 was found to be upregulated in RMS, where it targets PTEN [68]. These findings open up the opportunity to enhance existing therapeutic strategies. Therefore, future investigations should focus on the clinical application of miRNA modulation. The signaling pathways involved in the invasion and metastasis of rhabdomyosarcoma include RAS-MEK-ERK, Notch, Hedgehog and Wnt pathways, and Caveolin-1 signaling [9]. Also, paracrine factors and miRNAs contribute to the complex signaling networks that govern RMS biology, impacting tumor progression, metastasis, and response to therapy. Understanding these influences can help identify potential therapeutic targets and improve treatment strategies for RMS [9]. Ongoing research is trying to develop therapies that specifically target the PAX3-FOXO1 and PAX7-FOXO1 fusion proteins found in aRMS, which are critical for tumorigenesis [11]. Super enhancers (SEs) are large clusters of transcriptional enhancers that drive the expression of genes critical for cell identity and function [69]. SEs are often involved in oncogenic processes and are particularly important in PAX3-FOXO1 fusion-positive RMS. As a matter of fact, this fusion protein acts as a master regulator, promoting the formation of SEs that enhance the expression of target genes involved in muscle differentiation and tumorigenesis, such as MYOD, MYOG, and MYCN [70]. This creates a feedback loop that reinforces the expression of genes critical for RMS cell identity and survival. The presence of SEs associated with PAX3-FOXO1 and its target genes contributes to the aggressive nature of RMS. Therefore, understanding the role of PAX3-FOXO1 in establishing SEs opens up potential therapeutic avenues. Targeting the pathways involved in SE formation or the transcription factors associated with these enhancers may provide new strategies for treating fusion-positive RMS cases [70].

RMS cells can develop resistance to standard chemotherapeutic agents. Mechanisms of resistance include the upregulation of drug efflux pumps, the activation of survival pathways (e.g., PI3K/Akt/mTOR), and the bypass of targeted pathways. As a result, some tumors may continue to grow despite the treatment, leading to recurrence and metastasis. The complexity of cancer signaling pathways suggests that single-agent therapies may not be sufficient. Future studies may focus on combining PI3K-Akt inhibitors with other therapeutic modalities, such as chemotherapy, immunotherapy, or other targeted therapies (e.g., MEK/ERK inhibitors), to overcome resistance mechanisms and enhance anti-tumor efficacy. The continued exploration of small molecule inhibitors or monoclonal antibodies that specifically target components of the pathways activated by PAX3-FOXO1, such as IGF-1R or PI3K/mTOR, could lead to more effective treatments. The integration of targeted therapies like temsirolimus into RMS treatment regimens is crucial; however, the unfavorable results of these clinical trials underscore the complexity of effectively treating RMS, therefore emphasizing the need for further research and more personalized treatment strategies [42]. The expression of PAX3-FOXO1 fusion protein and the specific myogenic lineage from which the RMS tumors originate are significant factors contributing to the establishment of DNA methylation patterns. A very recent study identified distinct DNA methylation profiles associated with fusion-positive and fusion-negative RMS, providing new insights into tumor biology and emphasizing the potential of targeting the epigenetic landscape in RMS as a strategy for future therapeutic developments [71]. The development of advanced preclinical models (e.g., patient-derived xenografts or organoids) that more accurately reflect the tumor microenvironment and signaling interactions could contribute to a better evaluation of therapeutic candidates and combinations. Given the genetic origins of RMS, methods such as CRISPR-Cas9 or RNA interference to directly target the PAX3-FOXO1 fusion or components of the PI3K signaling pathway represent a forward-looking area of research that could offer breakthroughs in treatment.

The absence of reliable biomarkers able to predict treatment response or to monitor disease progression emphasizes the difficulty of tailoring therapies to individual patients. Identifying relevant biomarkers could help in selecting more effective cures and adjusting them based on disease evolution. Considering biomarkers and measuring circulating tumor DNA could significantly improve treatment monitoring and patient stratification by determining which patients are more likely to respond to specific therapies, thereby potentially optimizing the outcomes [44]. Overall, the future of rhabdomyosarcoma treatment lies in a more tailored, research-driven approach that incorporates molecular data and innovative methodologies for patient care. In this scenario, AI algorithms can analyze large datasets and identify new potential compounds or biomarkers that target specific pathways, such as the PI3K-Akt-mTOR, in RMS. Machine learning models can predict the efficacy and safety of these compounds, facilitating the identification of promising candidates for further testing [72]. By integrating multi-omics data, AI can help recognizing which patients are more likely to benefit from PI3K-Akt-mTOR inhibitors or other targeted therapies, and by analyzing patients’ data, it can recommend personalized treatment protocols based on individual tumor profiles. Future research needs to translate these novel insights into innovative approaches, which could offer a better outcome and a more personalized treatment option for the patients.

## 6. Conclusions

Rhabdomyosarcoma is a malignant pediatric tumor originating from skeletal muscle. Negative prognostic factors as well as drug resistance result in a poor prognosis for some patients. Therefore, it is necessary to broaden basic knowledge on tumor biology and to develop new therapeutic strategies. The PI3K-Akt-mTOR pathway is implicated in the origin and progression of several tumors, including RMS; thus, there is increasing interest in targeting its components. Many inhibitors have been recently developed and are currently under preclinical and clinical trials. However, drugs directed towards upstream elements are likely to fail in patients having downstream mutations. Moreover, targeting single components of the pathway may lead to toxicity or insufficient efficacy. The PI3K-Akt signaling is crucial for the proliferation, survival, migration, and invasion of RMS cells, making it a focal point for research and potential therapeutic interventions. Optimal efficacy may be achieved by using multiple PI3K-Akt-mTOR inhibitors in synergy or by combining them with other approaches, such as radiotherapy, chemotherapy, or therapies targeting other signal transduction pathways. Ongoing research on the mechanisms involved in uncontrolled RMS cell growth will provide basic knowledge to find new potential combination therapies, which will control the signaling pathway, the downstream effector molecules, and/or the altered epigenetic modifications to improve therapeutic options for RMS patients. The heterogeneity of tumors and the presence of multiple genetic alterations may complicate the effectiveness of PI3K inhibitors. Indeed, different cancers may respond differently based on their unique genetic and molecular profiles. These limitations highlight the need for combination therapies and a deeper understanding of the underlying biology of cancers to improve the efficacy of PI3K pathway inhibitors.

## Figures and Tables

**Table 1:** Latest clinical trials evaluating PI3K-Akt-mTOR pathway inhibitors in patients with RMS and other types of sarcomas or pediatric tumors. Many of these therapies are currently approved for other types of neoplasms or disease conditions.

Agent	Molecular Target	Approved for	Clinical Trial Phase (RMS)	Usage	Status	Clinical Trial Identifier
Everolimus	mTOR	Breast Cancer Pancreatic NETs NETs of the Lung NETs of the Gut Advanced RCC	Phase I Phase II Phase I/II	Monotherapy Monotherapy Combination	Completed Unknown Completed	NCT00187174 NCT01216839 NCT03245151
Lenvatinib	RTKs	Thyroid Carcinoma Hepatocellular Carcinoma Endometrial Carcinoma	Phase I/II	Combination	Completed	NCT03245151
Samotolisib	PI3K	N.A.	Phase II	Monotherapy	Completed	NCT03213678
Sirolimus	mTOR	Transplant medication S-LAM	Phase I Phase II	Combination Combination	Completed Recruiting	NCT01135563 NCT02574728
Temsirolimus	mTOR	Advanced RCC Mantle Cell Lymphoma	Phase I Phase I/II Phase II Phase II Phase III	Combination Combination Combination Combination Combination	Terminated Completed Completed Completed Active	NCT01204450 NCT00949325 NCT01614795 NCT01222715 NCT02567435

Abbreviations: NETs, Neuroendocrine Tumors; RCC, Renal Cell Carcinoma; S-LAM, Sporadic Lymphangioleiomyomatosis.

## Data Availability

No new data were created or analyzed in this study.
